# Pollen Development and Viability in Diploid and Doubled Diploid Citrus Species

**DOI:** 10.3389/fpls.2022.862813

**Published:** 2022-04-25

**Authors:** Jorge Lora, Andres Garcia-Lor, Pablo Aleza

**Affiliations:** ^1^Department of Subtropical Fruit Crops, Instituto de Hortofruticultura Subtropical y Mediterránea La Mayora (IHSM la Mayora-UMA-CSIC), Málaga, Spain; ^2^Centro de Citricultura y Producción Vegetal, Instituto Valenciano de Investigaciones Agrarias, Valencia, Spain

**Keywords:** *Citrus sinensis*, *C. clementina*, tetraploid, pollen performance, microsporogenesis, microgametogenesis, carbohydrate, breeding

## Abstract

Seedlessness is one of the most important agronomic traits in mandarins on the fresh fruit market. Creation of triploid plants is an important breeding strategy for development of new commercial varieties of seedless citrus. To this end, one strategy is to perform sexual hybridizations, with tetraploid genotypes as male parents. However, while seed development has been widely studied in citrus, knowledge of key steps such as microsporogenesis and microgametogenesis, is scarce, especially in polyploids. Therefore, we performed a study on the effect of ploidy level on pollen development by including diploid and tetraploid (double diploid) genotypes with different degrees of pollen performance. A comprehensive study on the pollen ontogeny of diploid and doubled diploid “Sanguinelli” blood orange and “Clemenules” clementine was performed, with focus on pollen grain germination *in vitro* and *in planta*, morphology of mature pollen grains by scanning electron microscopy (SEM), cytochemical characterization of carbohydrates by periodic acid–Shiff staining, and specific cell wall components revealed by immunolocalization. During microsporogenesis, the main difference between diploid and doubled diploid genotypes was cell area, which was larger in doubled diploid genotypes. However, after increase in size and vacuolization of microspores, but before mitosis I, doubled diploid “Clemenules” clementine showed drastic differences in shape, cell area, and starch hydrolysis, which resulted in shrinkage of pollen grains. The loss of fertility in doubled diploid “Clemenules” clementine is mainly due to lack of carbohydrate accumulation in pollen during microgametogenesis, especially starch content, which led to pollen grain abortion. All these changes make the pollen of this genotype unviable and very difficult to use as a male parent in sexual hybridization with the objective of recovering large progenies of triploid hybrids.

## Introduction

Polyploidy has created many possibilities in plant breeding, such as production of improved cultivars, because of increased vigor, larger organs, higher yield levels, increased tolerance to biotic and abiotic stresses, and production of seedless varieties (Sattler et al., 2016). In the case of citrus, triploidy is highly valuable because of production of seedless fruits, one of the most important characters appreciated by consumers in the fresh fruit market. Triploid plants give rise to aneuploid gametes with very low fertility (Otto and Whitton, 2000); they are generally sterile, although they can occasionally produce fruits with very few seeds and induce seed formation in other fruit varieties through cross-pollination. Therefore, production of triploid hybrids is an important breeding strategy for recovering new seedless citrus varieties (Ollitrault et al., 2008; Navarro et al., 2015). Citrus triploid hybrids can be recovered by 2x × 2x sexual hybridization and results from the union of an unreduced megagametophyte with haploid pollen (Aleza et al., 2010), or from interploid hybridization between diploid and tetraploid parents (Aleza et al., 2012a,b).

The vast majority of citrus plants are diploid (2n = 2x = 18), although aneuploids and euploids can occasionally be identified in seedlings, and triploid and tetraploid plants are the most frequent euploid variations (Lee, 1988). Citrus reproductive biology is very complex, and apomixis hampers breeding by sexual hybridization at diploid and polyploid levels. The apomictic mechanism in citrus is determined by adventitious embryony from nucellar cells (Frost and Soost, 1968; Koltunow, 1993), and it is present in most genotypes with the exception of citron (*Citrus medica* L.), pummelo [*C. maxima* (L.) Osb.], clementine (*C. clementina* Hort. ex Tan.) and some mandarin hybrids. Seeds of non-apomictic genotypes contain only one sexual embryo, whereas in apomictic genotypes there is one sexual embryo and multiple nucellar embryos genetically identical to the mother plant. Tetraploidization occurs with certain frequency in apomictic genotypes because of spontaneous duplication of chromosomes of nucellar cells (doubled diploids) (Lapin, 1937; Cameron and Frost, 1968; Aleza et al., 2011); however, this duplication does not occur in non-apomictic genotypes. Aleza et al. (2009) developed an efficient method for obtaining stable doubled diploid plants of non-apomictic citrus genotypes performing shoot tip grafting *in vitro* (Navarro and Juárez, 2007) and subsequently treating shoot tips with colchicine and oryzalin. Somatic hybridization by protoplast fusion is another technique that has been utilized in citrus to produce allotetraploid hybrids whether from apomictic or non-apomictic genotypes (Ollitrault et al., 2020). Natural or induced polyploidization can also produce several genetic and epigenetic changes, which lead to reduced viability of polyploid genotypes (Sattler et al., 2016). Citrus doubled diploid plants usually produce pollen grains with lower fertility than their original diploid genotypes (Frost and Soost, 1968; Aleza et al., 2012a), although pollen grain viability is sufficient for these types in order to be used as male parents in sexual interploid hybridization. Indeed, doubled diploid and allotetraploid somatic hybrids have been successfully used in citrus breeding programs as male parents (Starrantino and Recupero, 1981; Viloria and Grosser, 2005; Navarro et al., 2015; Ollitrault et al., 2020). Nevertheless, some doubled diploid plants produced either with antimitotic chemicals or by spontaneous chromosome doubling display very poor pollen performance, and there is not a comprehensive study on pollen ontogeny and performance of diploids and their related doubled diploid plants.

Pollen performance is highly dependent on correct pollen development (Lora et al., 2012; Lora and Hormaza, 2018), which is a complicated and highly conserved process in angiosperms that occurs in the anther (McCormick, 2004; Blackmore et al., 2007) and is of major importance for successful fertilization of plants. Microsporogenesis is a key step in plant life cycle and leads to microspore formation (haploid cells) (Blackmore and Knox, 1990; Nadot et al., 2008). Initially, a hypodermal archesporial cell divides and forms an inner layer of primary sporogenous cells and an outer layer of primary parietal cells, and further divides and forms somatic cell layers of the anther wall (binucleate tapetum, endothecium, and epidermis) (Frost and Soost, 1968). Primary sporogenous cells directly become microspore mother cells (MiMCs) or originate from a large number of sporogenous cells (Maheshwari, 1950). Diploid MiMCs increase in size concomitantly with accumulation of callose around the MiMC wall. MiMCs undergo meiosis to form a tetrad with haploid microspores; they are, at this moment, still surrounded by callose (Horner and Lersten, 1971; Bedinger, 1992; Schmidt et al., 2015). After microspores are released from the callose envelope, microsporogenesis is completed, and microgametogenesis starts, which gives rise to mature microgametophytes from microspores. Microspore volume grows rapidly after the release of the tetrad and is concomitant with callose degradation by the callase secreted by the tapetum (Scott, 2004; Lu et al., 2014). Proper timing of callase secretion is one of the critical moments in microsporogenesis, and its distortion causes male sterility (Worrall et al., 1992). Later, microgametogenesis begins and comprises events that change unicellular microspores into mature microgametophytes containing gametes or pollen grains. Thus, microspores develop as pollen grains by asymmetric division (mitosis I), and they contain a large vegetative cell that hosts a small generative cell (Holtzhausen and van der Schijff, 1977; Bedinger, 1992; Schmidt et al., 2015). When mitosis II takes place during microgametogenesis; for approximately 30% of species, it occurs before pollen germination and pollen is released as tricellular pollen at anther dehiscence. However, in the remaining 70% of angiosperms, mitosis II occurs after pollen germination in elongated pollen tubes, releasing bicellular pollen (Brewbaker, 1967), which is the case for citrus (Pok et al., 2015; Garavello et al., 2019).

Two key processes for proper pollen development in the anther are carbohydrate metabolism and dehydration (Pacini et al., 2006; Lora et al., 2012). In citrus, polysaccharides are present in different stages of pollen development, before and during MiMC meiosis, and at the end of pollen maturation, and serve as a source of energy (Hu et al., 2007). Carbohydrates not only protect male gametophytes against desiccation but also facilitate broad communication with stigma surface and maintain adequate pollen hydration (Herrero and Dickinson, 1981; Scott, 2004). A highly important issue during the formation of pollen grains is the formation of their cell wall, which is composed of an outer layer, or exine, and an inner layer, or intine (Blackmore et al., 2007). This process generally starts when individual microspores enlarge, and the nucleus divides, forming vegetative and generative nuclei, as in citrus (Frost and Soost, 1968). Microspores gradually take up substances provided by the tapetum, such as pectins and soluble carbohydrates, to store starch or sporopollenin, which is the main component of the exine. Sporopollenin is a mixture of biopolymers and protects the internal microenvironment of pollen (Hafidh et al., 2016). The intine is composed mainly of cellulose and pectins. The inner layer of the exine, the nexine, is composed of arabinogalactan proteins (AGPs), which have been described to be playing an important role in pollen development (Coimbra et al., 2007; Levitin et al., 2008). AGPs have also been observed in the intine in *Quercus suber* (Costa et al., 2015), the early divergent *Trithuria submersa* (Costa et al., 2013), and *Mangifera indica* (Lora and Hormaza, 2018). The exine is not uniformly distributed around pollen grains and is reduced in apertures where pollen tubes will grow. Formation of the pollen coat is completed at the end of microgametogenesis when residues of the degenerating tapetum are deposited on the surface of pollen grains (Quilichini et al., 2015). This coat is highly important for pollen adhesiveness, color, and aroma, and for interactions with stigmas and pollinators.

Although there are many studies on pollen development in flowering plants, there is little information on key steps in pollen grain development (microsporogenesis and microgametogenesis) that lead to male sterility in *Citrus* polyploids. In this study, we employed two citrus species at the diploid and tetraploid levels that are very interesting parents for breeding programs (Starrantino and Recupero, 1981; Navarro et al., 2015) and display different pollen performances, “Clemenules” clementine and blood orange [*C. sinensis* (L.) Osbeck]. “Clemenules” clementine, a natural hybrid between mandarin and sweet orange (Deng et al., 1996), is one of the most important and widely cultivated mandarin varieties in the Mediterranean Basin, especially in Spain, because of its excellent organoleptical properties, sexual characteristics, lack of furanocoumarins (FCs), and notable amount of flavonoids (Garcia-Lor et al., 2021). It presents good fertility at the diploid level but is not fertile at the tetraploid level. Blood orange varieties such as “Sanguinelli,” are rich in anthocyanins, which have been associated with important health benefits (Bonina et al., 2002; Kelebek et al., 2008; Titta et al., 2010). The red color in the pulp and/or peel of blood orange arose by insertion of a Copia-like retrotransposon adjacent to a gene encoding Ruby, an MYB transcriptional activator of anthocyanin production (Butelli et al., 2012).

In this study, we performed an integrated study on pollen development in these citrus species at the diploid and tetraploid ploidy levels, giving special attention to cell wall composition (pectins and AGPs) and carbohydrates, and with the main objective of shedding light on the stage of microsporogenesis or microgametogenesis in which these differences originate, and their cause and implications in triploid breeding programs based on sexual hybridization using doubled diploid male parents.

## Materials and Methods

### Plant Material

Diploid and doubled diploid adult trees of “Clemenules” clementine and “Sanguinelli” blood orange located at Instituto Valenciano de Investigaciones Agrarias (IVIA) in Moncada (Valencia, Spain) were used for experiments during the entire flowering period. A series of flower buds with 1-mm size were collected until anthesis. Doubled diploid “Clemenules” clementine was recovered by colchicine treatment (Aleza et al., 2009), and doubled diploid “Sanguinelli” blood orange was identified by spontaneous chromosome duplication in nucellar cells (Aleza et al., 2011).

### Establishment of the Appropriate Pollen Developmental Stage

To determine the stage of flower development in which pollen grains are already individualized for each genotype, we stained the flower buds and/or anthers with aniline blue. Flower buds of up to 6 mm in size were sliced using a sharp blade, stained with 0.1% aniline blue in 0.1 M K_3_PO_4_ buffer (Linskens and Esser, 1957), and then squashed. In larger flower sizes until anthesis, anthers were removed from flowers and then prepared as previously mentioned. Preparations were visualized using a Nikon ECLIPSE E800 epifluorescence microscope.

### Evaluation of *in vitro* Pollen Grain Germination

To evaluate *in vitro* pollen germination, at least 10 flowers per genotype were randomly collected from field-grown plants in the preanthesis stage. The anthers were removed from flowers with tweezers and placed in 35 mm Petri dishes, which were introduced in a desiccator over silica gel for 24–48 h. Pollen grains from fully dehisced anthers were distributed with a paintbrush on 5.5-cm Petri dishes containing MS culture medium (Murashige and Skoog, 1962) with 30 g/L sucrose and 8 g/L Bacto agar. The Petri dishes were placed on 9-cm Petri dishes with a piece of moist filter paper at 24 ± 1°C and in the dark for 24 h. Data were collected from three to four Petri dishes containing approximately 100 pollen grains each. The preparations were observed with a Leica microscope equipped with a Leica DFC490 camera. The pollen was scored as germinated when the length of the pollen tube exceeded the diameter of the pollen grain, and counted with the ImageJ2 software (Schindelin et al., 2015).

### Evaluation of *in planta* Pollen Grain Germination

To evaluate *in vivo* pollen germination and compare it with *in vitro* pollen germination, five flowers of a “Fortune” mandarin hybrid (*C. clementina* x *C. tangerina*) tree were hand-pollinated with pollen from the four male parents used in this study. “Fortune” mandarin was selected for its compatibility with diploid and doubled diploid clementines and blood oranges. *In planta* pollen grain germination was quantified directly on stigma surface where the pollen germination took place. Pollinated flowers were sampled 12–24 h after pollination. Pistils were fixed in an FAA solution (formalin, glacial acetic acid, and 70% ethanol; 1:1:18, v/v) (Johansen, 1940) and stored at 4°C for histological observations. The pistils were submerged three times in water for 1 h. Stigmas were stained for 24 h with 0.1% aniline blue in 0.1 M K_3_PO_4_ (Linskens and Esser, 1957). Then, the surface of the stigmas was sliced into sections and squashed to count pollen grain germination. The preparations were observed under a Leica MZ16FA stereomicroscope equipped with a GFP1 epifluorescence microscope and a Leica DC500 camera. Pollen grains were considered germinated when pollen tube length exceeded grain diameter. At least five stigmas for each genotype were prepared, and pollen grains were counted with the ImageJ2 software (Schindelin et al., 2015).

### Statistical Analysis

*In vitro* and *in planta* pollen grain germination from diploid and doubled diploid genotypes were analyzed by a proportion test (α = 0.05) considering binomial proportions. The null hypothesis (H_0_) for the test was considered when proportions are the same (P_1_ = P_2_), whereas the alternate hypothesis (H_1_) was that proportions are not the same (P_1_ ≠ P_2_) (Supplementary Table 1). A statistical analysis was performed using the Statgraphics Centurion XVII software (StatPoint Technologies, Inc., Warrenton, VA, United States), and making a comparison of two sample tabs, hypothesis testing, and selection of binomial proportions.

### Mature Pollen Grain Morphology

Scanning electron microscopy (SEM) was performed to observe the morphology of pollen grains in the mature stage. Anthers in the preanthesis stage were collected from three samples and freshly dried over silica gel for approximately 48 h. Pollen grains were directly attached to SEM stubs using adhesive carbon tabs and observed with a JSM-840 scanning electron microscope (JEOL) operated at 10 kV.

### Light Microscope Preparations

To study pollen development, whole bottom flowers were fixed until they reached a size of 6 mm. After this size was reached, anthers were extracted and fixed in 2.5% glutaraldehyde in 0.03 M phosphate buffer (Sabatini et al., 1963), dehydrated in an ethanol series, and embedded in Technovit 8100 (Kulzer & Co, Werheim, Germany).

For cytochemical study, semi-thin sections (2 μm) of the anthers in different developmental stages were stained with periodic acid–Schiff (PAS) and toluidine blue (Feder and O’Brien, 1968) for detection of insoluble polysaccharides and general histological observations. The sections were stained with 0.5% (w/v) periodic acid for 2 h, washed three times with water, and maintained in Schiff’s reagent in the dark for 1.5 h. After three washes with water, the sections were stained with aqueous 0.2% (w/v) toluidine blue (Feder and O’Brien, 1968).

### Inmunocytochemistry

The anthers were fixed in 4% paraformaldehyde in phosphate buffer saline (PBS) and left for 12 h at 4°C. Then, the anthers were dehydrated in an acetone series and embedded in Technovit 8100 (Kulzer). The samples were polymerized for 24 h at 4°C and sectioned (2 μm). The sections were placed in a drop of water on a slide, which was covered with a solution of 2% v/v 3-aminopropyltrietoxysilane in water (Sigma, St. Louis, MO, United States) and, finally, dried at room temperature (Satpute et al., 2005; Solís et al., 2008).

Inmunocytochemistry was performed as described by Solís et al. (2008) and Lora et al. (2009). For immunolocalization of methyl-esterified and unesterified pectins (Knox, 1997), we used the JIM7 and JIM5 rat monoclonal antibodies, respectively (Carbosource Service, University of Georgia, Athens, GA, United States). Presence of arabinogalactan proteins (AGPs) (Pennell et al., 1991) was identified using JIM8 rat monoclonal antibodies (Carbosource Service, University of Georgia, Athens, GA, United States), and of callose using anticallose mouse monoclonal antibody (Biosupplies, Parkville, VIC, Australia). To visualize nuclei, the sections were washed three times with PBS, stained with 4′,6-diamidino-2-phenylindole (DAPI, 0.1 mg/ml), and washed again with PBS. The sections were mounted in ProLong Gold Antifade Reagent (Invitrogen, Carlsbad, CA, United States), examined with a Leica DM2500 epifluorescence microscope with 470/525-nm filters, and photographed with a Leica DFC310 FX camera and the Leica Acquisition Station AF6000 E software.

## Results

### Phenotypic Characterization of Flowers and Pollen

The maximum flower size of the four genotypes varied, displaying differences between genotypes and ploidy levels. Diploid “Clemenules” reached a flower size of approximately 12 ± 2 mm (*n* = 15), whereas that of doubled diploid “Clemenules” was approximately 15 ± 2 mm (*n* = 14). Flowers of diploid and double diploid “Sanguinelli” were larger than those produced by “Clemenules,” reaching a length of 20 ± 2 mm (*n* = 11) and 27 ± 1 mm (*n* = 11) for diploids and doubled diploids, respectively.

As the four genotypes under study had different flower sizes, we performed a preliminary analysis of pollen development to select the best flower developmental stages for performing experiments to study microsporogenesis and microgametogenesis. To determine the presence of immature pollen grains, we stained with aniline blue dissected anthers of the four genotypes in different flower sizes. Aniline blue stains callose and helps to identify meiosis. At later developmental stage, callose is not apparent in the immature pollen grains. We determined the presence of immature pollen grains in diploid “Clemenules” at a flower bud size of 3 mm and a pollen cell area of 250 μm^2^ (*SD* = 37; *n* = 76), in doubled diploid “Clemenules” at a flower bud size of 6 mm with an area of 339 μm^2^ (*SD* = 50; *n* = 94), in diploid “Sanguinelli” in 8 mm long flower buds with an area of 274 μm^2^ (*SD* = 16; *n* = 40), and in doubled diploid “Sanguinelli” in 9-mm long flower buds with an area of 573 μm^2^ (*SD* = 97; *n* = 65) (Supplementary Figure 1).

**FIGURE 1:**
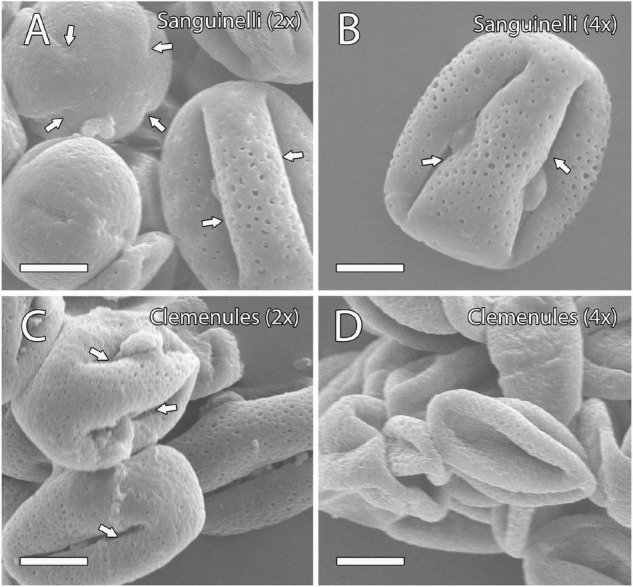
Observation of mature pollen grains of the four genotypes under study at anthesis by scanning electron microscopy (SEM). **(A–D)** Mature pollen grains showing a globose-ellipsoid shape in **(A)** diploid and **(B)** doubled diploid “Sanguinelli” and **(C)** diploid “Clemenules” with four aperture sites (arrows); however, the pollen grains of **(D)** doubled diploid “Clemenules” appeared shrunk. All scale bars: 10 μm. Diploid (2x) and doubled diploid (4x).

The pollen shape of these genotypes was further examined by SEM at anthesis when the two lobes of the anther are open and pollen grains are ready to pollinate. In doubled diploid “Clemenules,” the shape of the pollen appeared shrunk when compared to the globose-ellipsoid shape of pollen grains from diploid “Clemenules” and “Sanguinelli,” and doubled diploid “Sanguinelli” (Figure 1). The percentage of globose-ellipsoid pollen grains was 84% in diploid “Sanguinelli” (*n* = 114), 74% in doubled diploid “Sanguinelli” (*n* = 200), 87% in diploid “Clemenules” (*n* = 98), and 17% in doubled diploid “Clemenules” (*n* = 120). Pollen grains with four and five aperture sites have been previously reported in clementine (Al-Anbari et al., 2015), although in this study, we mainly observed pollen grains with four aperture sites (Figure 1).

We next studied the ability of these pollen types to germinate *in vitro* and *in vivo* on stigma surfaces. Taking into account all comparisons between two pollen grain germination percentages for the different genotypes, ploidy levels and *in vitro* versus *in vivo*, significant differences were observed on all pair combinations with few exceptions (Supplementary Table 1 and Supplementary Figure 2). The greatest differences were observed between doubled diploid “Clemenules” clementine, either *in vitro* or *in vivo*, and the other genotypes, rather than their ploidy level and way of cultivation (Table 1). Doubled diploid “Clemenules” clementine displayed lowest germination percentages both *in vitro* (0.3%) and *in vivo* (0.2%), with no statistical differences between them (Table 1 and *p*-value = 0.8874, Supplementary Table 1), highlighting their very low viability. On the contrary, diploid “Clemenules” clementine showed highest germination percentages, both *in vitro* and *in vivo* (Table 1). However, there were less statistical differences between diploid and doubled diploid pollen grains of “Sanguinelli” blood orange. For example, diploid and doubled diploid “Sanguinelli” pollen grains cultivated *in vivo* showed higher percentages than pollen grains cultivated *in vitro*.

**TABLE 1:** *In vitro* and *in vivo* pollen germination percentages of diploid and doubled diploid “Clemenules” clementine and “Sanguinelli” blood orange.

	Clem 2x		Clem 4x		Sang 2x		Sang 4x	
	*In vitro*	*In vivo*	*In vitro*	*In vivo*	*In vitro*	*In vivo*	*In vitro*	*In vivo*
Number of pollen grains counted	368	249	345	527	106	154	561	128
Number of pollen grains germinated	297	139	1	1	13	49	76	28
Germination percentage	80.7	55.8	0.3	0.2	12.7	31.8	13.6	21.9

Taken together, in all results of the phenotypic characterization of flowers and pollen grains, flower size was bigger in blood oranges than in clementines, and in doubled diploids than in their corresponding diploid genotypes, but more relevant was the anomalous shape of pollen grains of doubled diploid “Clemenules” and its very low viability in comparison with the other genotypes regardless of ploidy level and way of growing (*in vitro* vs. *in vivo*).

### Microsporogenesis and Release of Microspores

**FIGURE 2:**
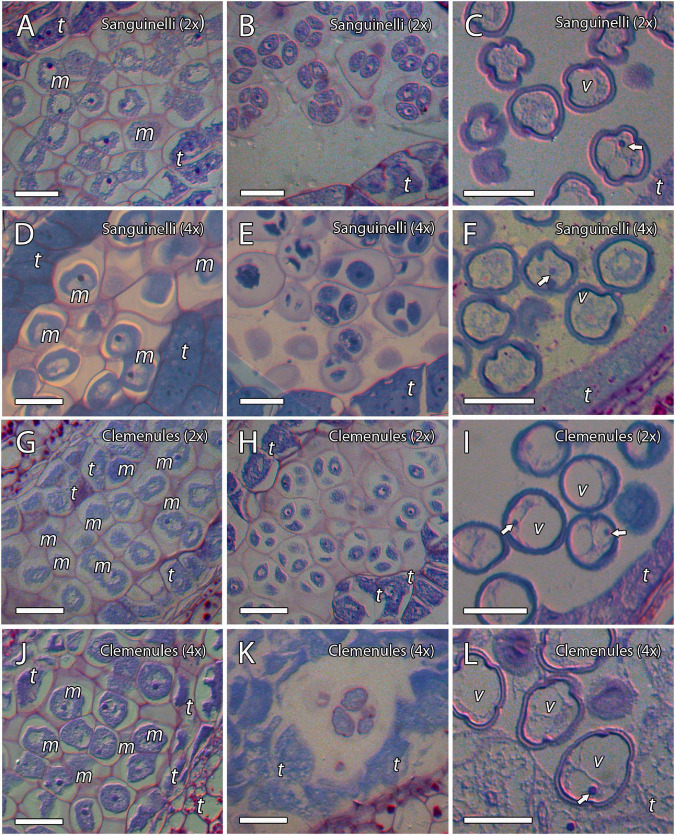
Microsporogenesis of diploid and doubled diploid “Sanguinelli” blood orange and “Clemenules” clementine. Sections were stained with periodic acid-Schiff reagent (PAS) and toluidine blue. **(A,D,G,J)** Microspore mother cells surrounded by the tapetum. **(B,E,H,K)** Microspores undergoing meiosis and cytokinesis. **(C,F,I,L)** Individualized microspores with cytoplasmic vacuolization and the nucleus displaced laterally (white arrows). All scale bars = 25 μm. Tapetum (t), microspore mother cells (m), vacuole (v), diploid (2x), and doubled diploid (4x).

To evaluate the cause behind the extremely low pollen germination and the presence of pollen shrinkage in doubled diploid “Clemenules” clementine compared to the other genotypes evaluated, we performed an integrative study on pollen development by cytochemical characterization and immunolocalization. We first studied the early stages of pollen development, microsporogenesis, and the release of microspores by staining semi-thin resin sections with periodic acid-Schiff (PAS) reagent and toluidine blue for general histological observation, which also revealed the level of carbohydrate reserves. MiMCs were clearly observed in 3- to 6-mm long flower buds and were surrounded by the tapetum, which formed the innermost layer of the anther wall (Figures 2A,D,G,J). The cell area of the MiMCs was generally smaller in the diploid genotypes than in the doubled diploid genotypes. Diploids “Sanguinelli” and “Clemenules” showed an average MiMC area of 272 μm^2^ (*SD* = 46; *n* = 90) and 226 μm^2^ (*SD* = 30; *n* = 79), respectively, whereas at the tetraploid level, MiMC areas were 347 μm^2^ (*SD* = 77; *n* = 118) and 280 μm^2^ (*SD* = 37; *n* = 96) for “Sanguinelli” and “Clemenules,” respectively. Subsequently, MiMCs of the four genotypes underwent meiosis by simultaneous cytokinesis and were surrounded by the rest of the MiMC wall and insoluble carbohydrates (Figures 2B,E,H,K). After the release of unicellular microspores, the microspores increased in size, which caused vacuolization and laterally displaced the nucleus (Figures 2C,F,I,L).

**FIGURE 3:**
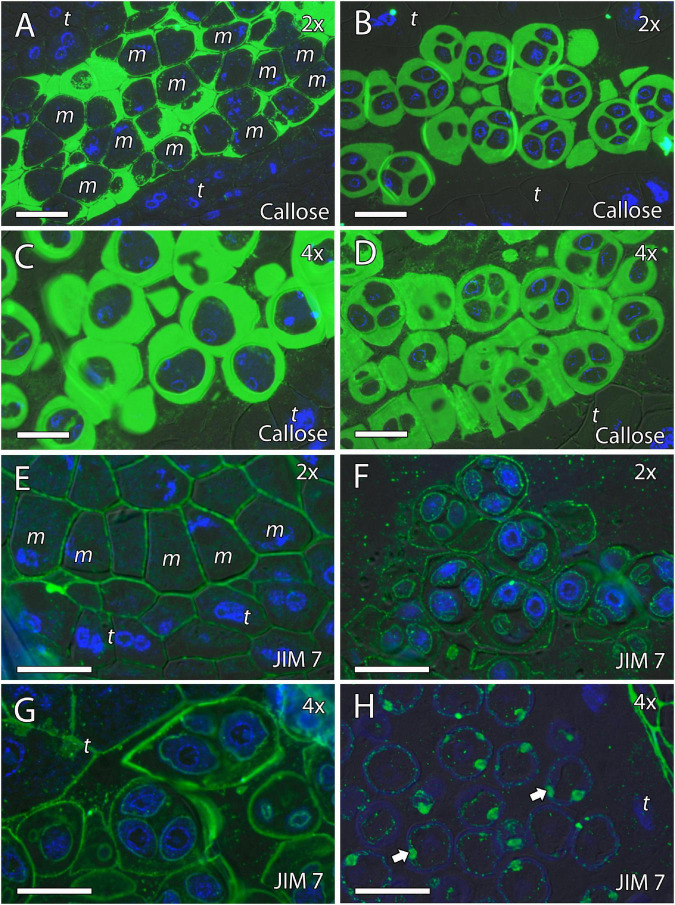
Inmunolocalization of callose and pectins during microsporogenesis in diploid and doubled diploid “Sanguinelli” blood orange. Monoclonal antibodies used: **(A–D)** anti-callose against callose and **(E–H)** JIM7 against methyl-esterified pectins. **(A,C)** Anti-callose labeling around MiMCs. **(B,D)** Callose prominently present around four microspores during simultaneous cytokinesis. **(E)** Pectins present in the MiMC wall. **(F,G)** Pectins present surrounding the microspores and in the early microspore wall. **(H)** Pectins observed in the aperture sites (arrows). All scale bars = 25 μm. MiMC (m), tapetum (t), diploid (2x), and doubled diploid (4x).

**FIGURE 4:**
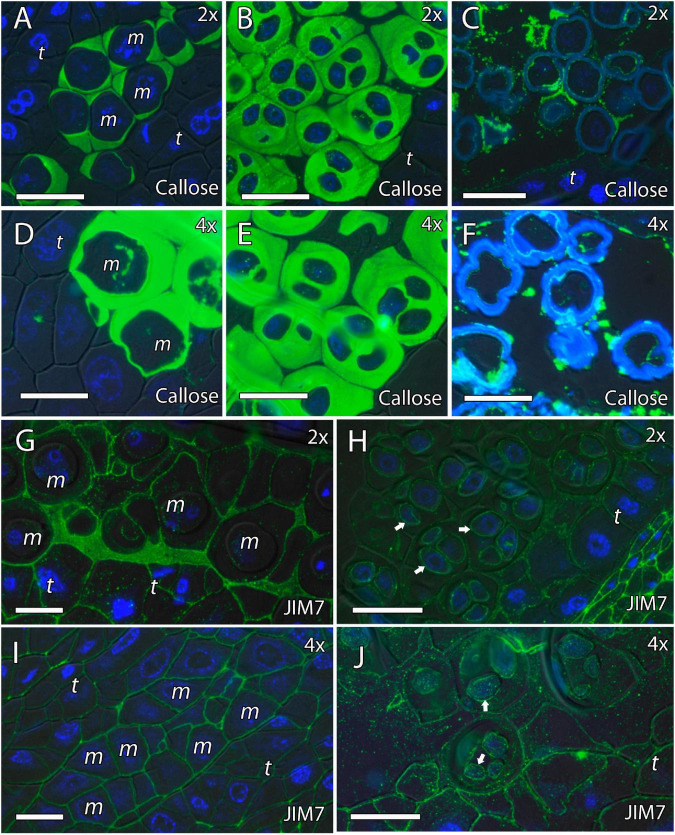
Inmunolocalization of callose and pectins during microsporogenesis in diploid and doubled diploid “Clemenules” clementine. Monoclonal antibodies used: **(A–F)** anti-callose against callose and **(G–J)** JIM7 against methyl-esterified pectins. **(A–C,G,H)** Diploid “Clemenules” and **(D–F,I,J)** doubled diploid “Clemenules”. **(A,D)** Anti-callose labeling around MiMCs. **(B,E)** Anti-callose labeling around the four microspores during simultaneous cytokinesis. **(C,F)** Callose was still present after microspore tetrad release. **(G,I)** JIM7 labeling on the MiMC wall. **(H,J)** JIM7 labeling was still present on the MiMC wall and during early microspore wall formation (arrows). All scale bars = 25 μm. Tapetum (t), MiMC (m), diploid (2x), and doubled diploid (4x).

**FIGURE 5:**
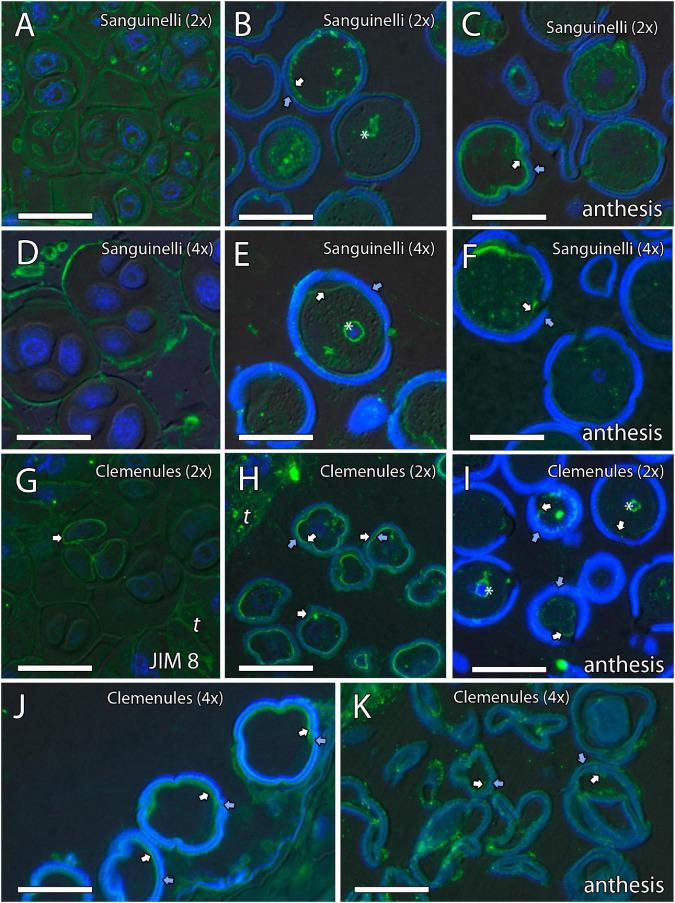
Inmunolocalization of arabinogalactan proteins (AGPs) during pollen development in diploid and doubled diploid “Sanguinelli” blood orange and “Clemenules” clementine. Monoclonal antibody used: JIM8 against AGPs. **(A,D)** Tetrads that retained the wall of microspore mother cells (MiMCs) and reacted positively to JIM8 in **(A)** diploid and **(D)** doubled diploid “Sanguinelli”. **(B,E)** Pollen grains showing JIM8-specific labeling in the intine (white arrow) and the wall of the generative nucleus (asterisk) after mitosis I and vacuolization in **(B)** diploid and **(E)** doubled diploid “Sanguinelli”. **(C,F)** Mature pollen grains at anthesis with JIM8 partial reaction in the intine (white arrow) and cytoplasm in **(C)** diploid and **(F)** doubled diploid “Sanguinelli.” **(G)** Tetrads showing weak JIM8-specific labeling on the microspore cell wall (white arrow) in diploid “Clemenules”. **(H,J)** Individualized microspores where JIM8 reacted in the intine (white arrows) in **(H)** diploid and **(J)** doubled diploid “Clemenules”. **(I,K)** Mature pollen grains showing weak JIM8 reaction in the intine (white arrows) of the pollen grains at anthesis in **(I)** diploid and **(K)** doubled diploid “Clemenules”. **(I)** Wall of the generative nuclei (asterisks) reacted to JIM8 in diploid “Clemenules”. The exine is shown in blue (autofluorescence, blue arrows). All scale bars = 25 μm. Tapetum (t), diploid (2x), and doubled diploid (4x).

Because cell wall composition is highly correlated with cell shape (Chebli and Geitmann, 2017), we also studied the localization of some principal cell wall components. To this end, we revealed methyl-esterified and unesterified pectins using the antibodies JIM7 and JIM5, respectively. In addition, we observed the presence of callose using antibodies against callose and arabinogalactan proteins (AGPs) with the JIM8 antibody. Inmunocytochemical studies revealed callose around the MiMC. After meiosis, callose was also observed around the resulting microspore tetrad in the four genotypes (Figures 3A–D, 4A,B,D,E). The MiMC was well-distinguished from the surrounding tapetum cells by its bigger size. The cell wall of the MiMC and the early microspores reacted positively with JIM7 (Figures 3E–G, 4G,I,H,J), JIM 5 (Supplementary Figures 3A,E,B,F, 4A–D), and JIM8 (Figures 5A,D,G) in the four genotypes. During the release of microspores, the callose gradually disappeared (Figures 4C,F). Interestingly, pectins that reacted to JIM7 and JIM5 were observed in aperture sites of the four genotypes (Figure 3H and Supplementary Figures 3C,G, 4B). With the JIM8 antibody used to recognize AGPs, a very faint reaction was observed in the tetrads, whereas the reaction was very clear in the intine of individualized microspores in all the genotypes (Figures 5A–K).

In summary, PAS and toluidine blue observations revealed proper carbohydrate reserves, and immunocytochemistry showed that the MiMC in the four genotypes was surrounded by a cell wall formed by callose (detected with anticallose antibody), pectins (JIM7 and JIM5 antibodies), and AGPs (JIM8 antibody), and was clearly distinct from somatic tapetum cells. The immunodetection of the cell wall components was similar in all genotypes during microgametogenesis. The main difference observed during the early stages of pollen development of the four genotypes was that MiMC cell area was larger in the doubled diploid genotypes.

### Formation of Pollen Cell Wall

**FIGURE 6:**
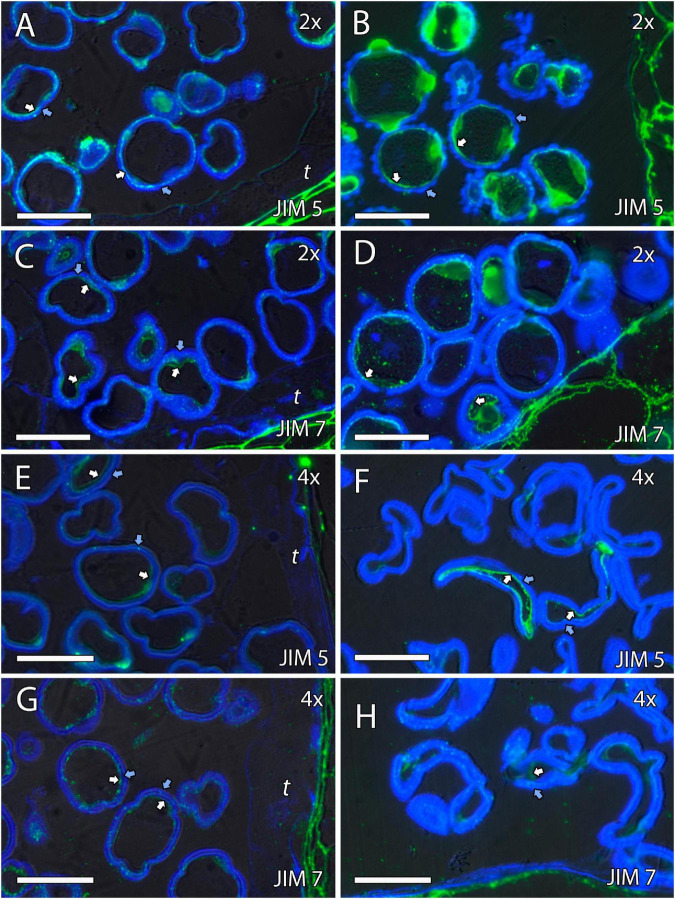
Immunolocalization of pectins during microgametogenesis in diploid and doubled diploid “Clemenules” clementine. Monoclonal antibodies used: **(A,B,E,F)** JIM5 against unesterified pectins and **(C,D,G,H)** JIM7 against methyl-esterified pectins. **(A,C,E,G)** JIM5- and JIM7-specific labeling (white arrows) was weakly observed in the intine of the microspore wall and in the aperture sites in the vacuolated stage in **(A,C)** diploid and **(E,G)** doubled diploid “Clemenules”. Unesterified (JIM5) **(B)** and methyl-esterified (JIM7) **(D)** pectins (white arrows) were observed in the intine and aperture sites of pollen grains at anthesis in diploid “Clemenules” clementine. Pollen grains of doubled diploid “Clemenules” shrank at anthesis, showing unesterified (JIM5) **(F)** and methyl-esterified (JIM7) **(H)** pectins (white arrows) in the intine. The exine was observed in blue due to autofluorescence, and it is indicated with blue arrows. All scale bars = 25 mm. Tapetum (t), diploid (2x), and doubled diploid (4x).

**FIGURE 7:**
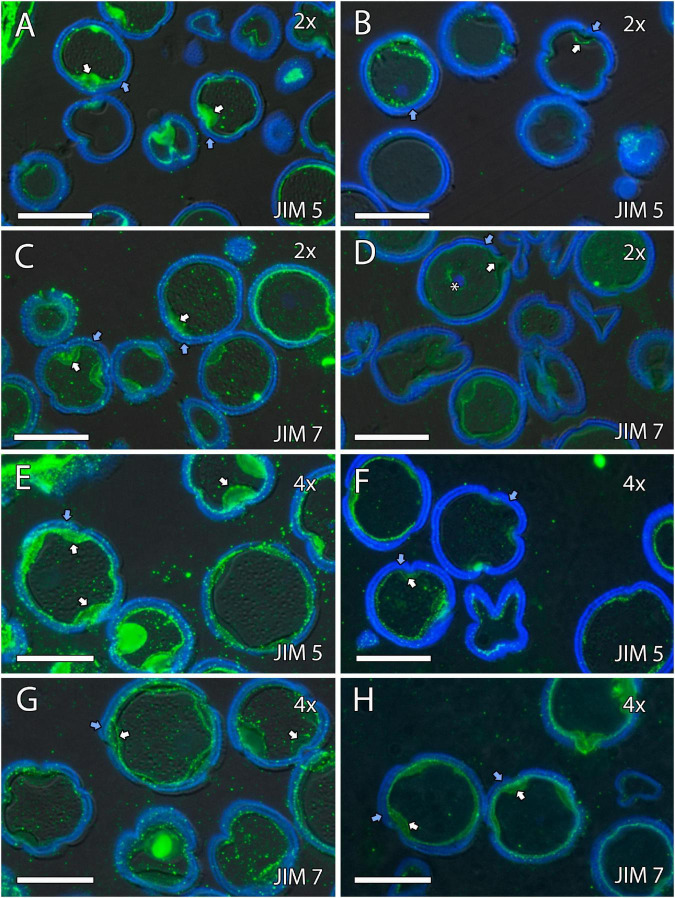
Immunolocalization of pectins during microgametogenesis in diploid and doubled diploid “Sanguinelli” blood orange. Monoclonal antibodies used: **(A,B,E,F)** JIM5 against pectins and **(C,D,G,H)** JIM7 against methyl-esterified pectins. **(A,C,E,G)** JIM5 **(A,E)** and JIM7 **(C,G)** labeling (pectins) was observed in the aperture sites (white arrows) and in the intine after mitosis I and vacuolization. Similar JIM5 **(B,F)** and JIM7 **(D,H)** labeling was observed in the intine and wall of the generative nucleus (asterisk in **D**) of pollen grains at anther dehiscence. The exine was observed in blue (autofluorescence, blue arrows). All scale bars = 25 mm. Tapetum (t), diploid (2x), and doubled diploid (4x).

We continued the inmunohistochemical study on the microgametogenesis stage. When microspores increased in size and underwent vacuolization, pectins were faintly detected in the intine and aperture sites of diploid and doubled diploid “Clemenules” (Figures 6A,C,E,G and Supplementary Figures 3D,H) but were more strongly detected at anthesis (Figures 6B,D,F,H). Similarly, the intine of diploid and doubled diploid “Sanguinelli” blood orange, including the apertures, reacted to JIM5 and JIM7 (pectins) after mitosis I (Figures 7A,C,E,G) and at anthesis (Figures 7B,D,F,H). AGP-specific labeling was faintly observed in the intine of all the genotypes at anthesis (Figures 5C,F,I,K), although it was slightly higher in diploid and doubled diploid “Sanguinelli” blood orange. Interestingly, AGPs were observed around generative cells in diploid and doubled diploid “Sanguinelli” blood orange and diploid “Clemenules” clementine (Figures 5B,E,I). To visualize the cell nuclei, the sections were counterstained with DAPI, showing the nuclei in blue, which confirmed generative cells in the abovementioned genotypes but not in doubled diploid “Clemenules.” While pectins and AGPs were similarly observed in the inner layer of the microspores and pollen grains of all the genotypes, the pollen shape of doubled diploid “Clemenules” clementine was clearly different.

### Microgametogenesis

**FIGURE 8:**
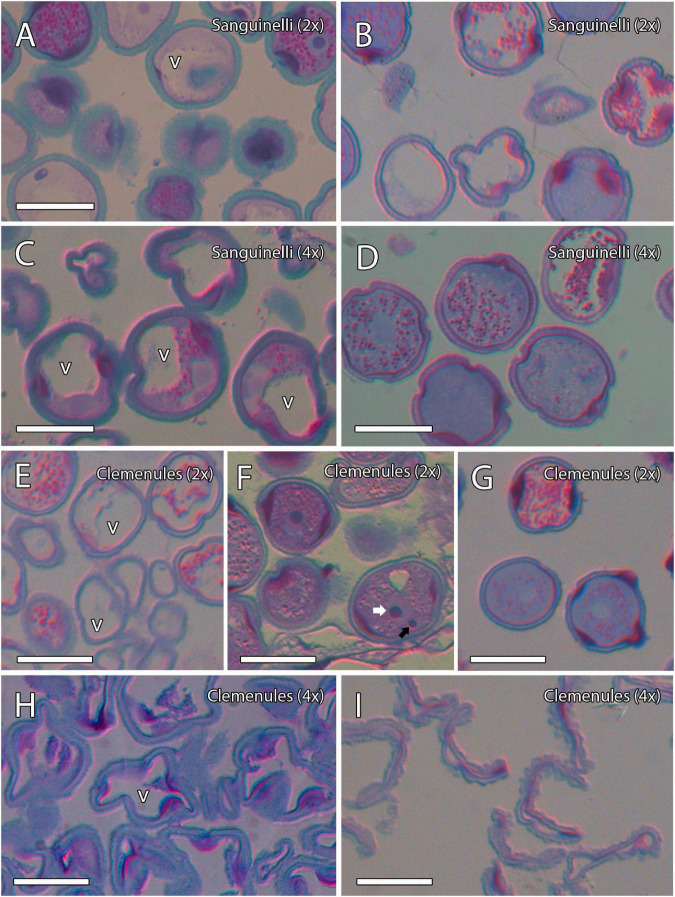
Microgametogenesis of diploid and doubled diploid “Sanguinelli” blood orange and “Clemenules” clementine. Sections were stained with periodic acid-Schiff reagent (PAS) and toluidine blue. **(A,C,E,F)** Starch accumulation (grains in pink color) in vacuolate microspores and concomitant mitosis I in **(A)** diploid “Sanguinelli” and **(E,F)** “Clemenules” and **(C)** doubled diploid “Sanguinelli”. **(F)** Pollen grain showing central vegetative nucleus (white arrow) and lateral generative nucleus (black arrow) in diploid “Clemenules” clementine. **(H)** Starch grains were not observed in vacuolate microspores in doubled diploid “Clemenules”. **(B,D,G)** Diploid “Sanguinelli” **(B)** and “Clemenules” **(G)** and doubled diploid “Sanguinelli” **(D)** show starch-less and starch-containing pollen grains at anthesis. **(I)** Doubled diploid “Clemenules” clementine shows pollen grains without starch grains and shrinkage at anthesis. All scale bars = 25 μm. Diploid (2x), doubled diploid (4x), and vacuole (v).

To further evaluate the difference between the shrunken pollen grains of doubled diploid “Clemenules” clementine and those of the remaining genotypes, we performed a cytochemical characterization of microgametogenesis, paying special attention to carbohydrates that are essential for internal turgor pressure (Pacini et al., 2006). Following vacuolization of the microspore cytoplasm, the unicellular microspores of diploids “Sanguinelli” and “Clemenules” and doubled diploid “Sanguinelli” underwent mitosis and began to accumulate starch grains, and their vacuoles decreased in size (Figures 8A,C,E,F). However, starch grains were almost not observed in the microspores of doubled diploid “Clemenules” (Figure 8H). The microspores underwent first mitosis and became bicellular pollen grains in diploid and tetraploid “Sanguinelli” and diploid “Clemenules” (Figure 8F), but first mitosis was not observed in doubled diploid “Clemenules.” The starch granules vanished in some pollen grains, and starchy and starchless pollen grains were observed at anther dehiscence in diploid and doubled diploid “Sanguinelli” and diploid “Clemenules” (Figures 8B,D,G). During microgametogenesis, pollen development followed a globose-ellipsoid shape in all genotypes except for doubled diploid “Clemenules,” which showed loss of area and a wrinkled shape (Figures 8H,I). Thus, while diploid and tetraploid “Sanguinelli” and diploid “Clemenules” showed pollen grain cell areas of 437 μm^2^ (*SD* = 61, *n* = 50), 636 μm^2^ (*SD* = 92, *n* = 19) and 412 μm^2^ (*SD* = 37, *n* = 35), respectively, the cell area of doubled diploid “Clemenules” was 95 μm^2^ (*SD* = 75, *n* = 50), being the only one smaller than in the previous stages of development, MiMC (280 μm^2^), and immature pollen grains (339 μm^2^).

As a conclusion of the results observed during microgametogenesis, we have stated that after the increase in size and vacuolization of the microspores and before mitosis I, doubled diploid “Clemenules” clementine showed drastic differences in shape, cell area, and starch hydrolysis. Thus, the microspores of doubled diploid “Clemenules” clementine increased in size but were not able to form starch grains, undergo mitosis, or expand their cell area, resulting in shrinkage of the pollen grains.

## Discussion

### External Morphological Changes Induced by Polyploidy

The most extensive consequence of polyploidy in plants is the increase in cell size, also known as the Gigas effect, caused by a large number of gene copies (Sattler et al., 2016). In this study, significant differences in flower size were observed in relation to ploidy level. The doubled diploid genotypes displayed larger flower sizes, pollen areas, and MiMC areas than the diploid genotypes. These kinds of differences have been previously observed in leaves, fruits, seeds, and roots of diploid and doubled diploid citrus plants (Romero Aranda et al., 1997; Ruiz et al., 2020), in floral organs of haploid and diploid clementines (Aleza et al., 2009), and in other species such as watermelon (Jaskani et al., 2005) and orchids (Gross and Schiestl, 2015). The diploid “Clemenules” clementine and diploid and doubled diploid “Sanguinelli” blood orange pollen grains were tetracolporate and pentacolporate and had a globose-ellipsoid shape, as were also observed by Al-Anbari et al. (2015). However, polyploidy in “Clemenules” clementine results in shrinkage of the majority (83%) of pollen grains.

### Pollen Performance Differed Across Ploidy Levels

Differences in pollen size are also reflected in differences in pollen performance (Souza-Pérez et al., 2021). We observed that pollen germination rates, both *in vitro* and *in vivo*, were higher in diploid “Clemenules” clementine than in the doubled diploid, whereas less differences were displayed for diploid and doubled diploid “Sanguinelli” blood orange. Aleza et al. (2012a) examined pollen germination rates of several doubled diploid genotypes that were similar to or less than those of corresponding diploid genotypes, as in our study. However, despite lower pollen germination rate, the remaining pollen fertility is usually adequate for successful controlled hand pollination (Ollitrault et al., 2008; Aleza et al., 2012a), which is the case for doubled diploid “Sanguinelli” blood orange but not for doubled diploid “Clemenules” clementine. In fact, in the framework of our triploid breeding program (Navarro et al., 2015), we recovered more than 400 triploid hybrids from more than 1,100 pollinated flowers, with doubled diploid “Sanguinelli” blood orange as the male parent, whereas only four triploid hybrids were recovered from more than 300 pollinations when doubled diploid “Clemenules” clementine was the male parent. These results clearly demonstrate that the ploidy level of pollen grains affects their viability, being higher in the diploid genotypes than in the doubled diploid analyzed genotypes. Moreover, if we compared the pollen performance of doubled diploid genotypes, a clear difference was observed. Doubled diploid “Sanguinelli” blood orange pollen grains were able to pollinate and recover triploid hybrids, where a double diploid “Clemenules” clementine had very few or no viable pollen and cannot be used as a male parent in sexual hybridization for the production of large progenies of triploid hybrids. Thus, the ploidy level of pollen grains is not the only factor that reduces their viability, since doubled diploids “Sanguinelli” blood orange and “Clemenules” clementine display the same ploidy but have different pollen performances, suggesting that there is a difference between them during the process of pollen development in either microsporogenesis or microgametogenesis.

### Similar Microsporogenesis Processes in Diploid and Doubled Diploid Genotypes

Adequate male germline development is a prerequisite for proper pollen performance. In the four genotypes studied, the initial development of the male germline, microsporogenesis, started normally with formation of archesporial cells, followed by MiMC development, and release of microspores.

During the last step of MiMC development and meiosis, there is intense crosstalk between the MiMC and the surrounding tapetum (Lora and Hormaza, 2020). Diploid MiMCs are surrounded by callose, an important polysaccharide that acts as a physical molecular filter (Tucker et al., 2001) and forms a tetrad with haploid microspores through meiosis. These microspores are individualized by the formation of the cell wall, but they remain surrounded by the callose of the original mother cell, similar to that which usually occurs in flowering plants (Bedinger, 1992; Schmidt et al., 2015). Callose plays a key role in the process of microsporogenesis. For example, in *Arabidopsis*, rice, and oat, different mutated genes leading to abnormal or defective callose walls and inviable mature pollen grains have been identified (Chen and Kim, 2009; Shi et al., 2015; Xiao et al., 2016). It seems that in our case, there was no failure in callose secretion due to the presence of callose around the MiMC that gradually disappeared during the release of microspores in the four genotypes studied.

Additionally, the unesterified and esterified pectins detected with the JIM 5 and JIM 7 antibodies, respectively, and the AGPs detected with the JIM 8 antibody were clearly identified on the MiMC wall during microsporogenesis. Similar results have been observed around the MiMC during meiosis in other species, such as *Annona* (Lora et al., 2009, 2014), *Mangifera indica* (Lora and Hormaza, 2018), *Arabidopsis thaliana* (Coimbra et al., 2007), *Beta vulgaris* (Nothnagel et al., 2001; Majewska-Sawka and Rodriguez-Garcia, 2006), and *Quercus suber* (Costa et al., 2015). Therefore, the cell wall of the MiMC seems to have developed properly in the four genotypes. Thus, in our study, all the genotypes seemed to present normal meiosis and cytokinesis, even doubled diploid “Clemenules” clementine. However, pollen development in this genotype was critically compromised during microgametogenesis, leading to its abortion.

### Differences Observed During Microgametogenesis

After microspores are released from the tetrad, they mature as individual pollen grains by asymmetric division (mitosis I). They start to develop the inner (intine) and outer (exine) pectocellulosic layers of their cell walls, in which the tapetum plays an essential role in the deposition of components for exine formation (Lora and Hormaza, 2020). In *Limonium* sp. (Róis et al., 2012), microspores of tetraploids only develop until the ring-vacuolate stage because of the absence of typical exine patterns, pointing to sporophytic defects. In a recent review on lipid metabolism (Wan et al., 2020), genes involved in anther and pollen development in different species were studied. Lipids are mainly synthesized in the anther tapetum and can be transported to the surface of the pollen and other anther wall layers (e.g., epidermis, endothecium, and middle layer); these lipids are critical for promotion pollen wall formation (e.g., sporopollenin to form exine) (Piffanelli et al., 1997; Blackmore et al., 2007; Parish and Li, 2010). On the other hand, pollen coat lipids are important for pollen hydration in later stages (Wolters-Arts et al., 1998). In our study, lipids were not apparently involved in pollen abortion in doubled diploid “Clemenules” clementine, because exine formation seemed to be normal, and the pollen cell wall formed, creating individualized microspores and subsequent pollen grains.

Signals of the pectins detected with JIM5 and JIM7 were observed during microgametogenesis in the aperture sites and in the intine of the microspores; these signals were stronger in later stages. A similar labeling pattern of intine has been observed in other species (Lora et al., 2009; Lora and Hormaza, 2018), and although callose deposition is usually observed in the aperture site (Lora et al., 2009; Lora and Hormaza, 2018), it has not been observed in diploid and doubled diploid genotypes of “Clemenules” clementine and “Sanguinelli” blood orange.

In early microgametogenesis, we observed AGPs in the intine of all genotypes, as was observed by Abreu and Oliveira (2004). In *Arabidopsis*, AGPs were present in the inner layer of the exine and the nexine, and in young microspores (Jia et al., 2015), and have been shown to be essential in pollen development. Levitin et al. (2008) showed that loss of function of the *AGP6* and *AGP11* genes in *Arabidopsis* led to reduced fertility due to pollen tube growth inhibition and inability to release pollen grains from the mature anther. In contrast, Coimbra et al. (2009) showed that in the double mutant *agp6agp11* in *Arabidopsis*, collapse of pollen grains started in the stage of young microspores and was accompanied by extensive shrinkage of the cytoplasm and some other adverse effects. Interestingly, we also observed AGPs around the generative cell in diploid “Clemenules” and in diploid and doubled diploid “Sanguinelli” that has also been observed in other angiosperms such as *A. thaliana* (Coimbra et al., 2009) and *Q. suber* (Costa et al., 2015). However, similar AGP labeling was not detected in doubled diploid “Clemenules” clementine, which may be a consequence of the impairment of mitosis I. Conversely, the lack of AGPs cannot be excluded as a cause of mitosis I impairment. These AGPs may be present in the final stages of microgametogenesis in these fertile genotypes, because their role as interactors is essential during crosstalk between the pollen tube and female reproductive tissues (Pereira et al., 2016).

During microgametogenesis, unicellular microspores and subsequent bicellular pollen grains also absorb carbohydrates and soluble sugars from the tapetum that can be metabolized, converted to other molecules, used for formation of the intine, or stored (Pacini, 1996). Carbohydrates are essential for maintaining the internal turgor pressure of pollen and, thus, regulating water balance, which is crucial for maintaining pollen viability over time and essential for dispersal at anthesis (Pacini et al., 2006). Starch content has been deeply studied in different species as an indicator for mature viable pollen (Wang et al., 2004). Interestingly, starch grains were almost not observed in doubled diploid “Clemenules.” Although microspores underwent vacuolization, vacuolization was not concomitant with accumulation of starch grains. Moreover, mitosis I was not observed. Thus, further microspore development was suspended, and we only observed shrinkage of the microspores after vacuolization. On the other hand, the microspores of diploid and doubled diploid “Sanguinelli” blood orange and diploid “Clemenules” clementine underwent the first and second mitoses and became bicellular pollen grains, which is in agreement with the results of previous studies on *Citrus* (Pok et al., 2015; Garavello et al., 2019).

The crucial role of starch grains in pollen development has also been observed in a seedless mutant of “Ougan” mandarin (*C. suavissima* Hort. Ex Tan.) (Hu et al., 2007). The authors identified less starch grains and lipids in early mature pollen grains of the mutant in comparison with the original mandarin. Starch biosynthesis during the final steps of pollen maturation is critical (Datta et al., 2002). In maize (Wen and Chase, 1999), unviable pollen was associated with starch deficiency in S-type cytoplasmic male sterility (S-CMS). In sorghum, Datta et al. (2001) observed changes in gene expression between starch-filled (fertile) and starch-deficient (sterile) pollen grains during transition from vacuolated microspores to immature pollen grains.

### Implications for Citrus Triploid Breeding Programs

Diploid by tetraploid sexual hybridization is one strategy that allows to recover large populations of citrus triploid hybrids (Viloria and Grosser, 2005; Navarro et al., 2015; Ollitrault et al., 2020). For this strategy to be efficient, tetraploid male parents must display enough pollen performance to achieve sexual matting. The pollen viability of doubled diploid plants produced by spontaneous chromosome doubling is similar or less than that of corresponding diploid genotypes, as was previously observed by Aleza et al. (2012a) and stated in this study for “Sanguinelli” blood orange. Nevertheless, doubled diploid “Clemenules” clementine, recovered after colchicine treatment, showed very less or practically null pollen viability. In the framework of our triploid breeding program, we obtained a large number of doubled diploid plants from non-apomictic genotypes using antimitotic chemicals, and it is important to point out differences in pollen performance and viability between them. For example, stable doubled diploid plants of “Chandler” pummelo and “Moncada” (Garavello et al., 2020), “Temple,” and “Encore” mandarins have good pollen performance, whereas other doubled diploid mandarin varieties like “Wilking,” “Marisol,” and “Fina” clementines display less pollen performance. In other crop species like flax, sunflower, barley, and cotton (Luckett, 1989; Yemets and Blume, 2008; Ahmadi and Ebrahimzadeh, 2020), it has been observed that colchicine can cause chromosome loss or rearrangement and gene mutations, which could affect the viability of pollen grains. However, more research is needed to shed light on this matter.

The genotypes studied in this article have different reproductive characteristics. “Clemenules” clementine is non-apomictic, and doubled diploid plants can be used as female parents in 4x × 2x sexual hybridization with high efficiency (Aleza et al., 2012b), whereas “Sanguinelli” blood orange is apomictic, and its utilization as a female parent is not efficient for the production of large progenies of triploid hybrids, directing interest in it as a male parent for 2x × 2x and 2x × 4x crosses at the diploid and tetraploid ploidy levels, respectively. The genetic structure of diploid gamete populations and, particularly, parental heterozygosity restitution (PHR) are driven by diploid gamete origin. For double diploids resulting from somatic chromosome doubling of diploid varieties, PHR theoretically leads to 66% restitution of the heterozygosity of a diploid that originates from a tetraploid (Sanford, 1983; Aleza et al., 2016), although slight differences have been observed among linkage groups in doubled diploid “Clemenules” clementine (Aleza et al., 2016). Furthermore, depending on the linkage group in which a gene controlling an eventual trait of interest is located, genetic regulation of the trait, and strategy of crossing, different segregation patterns can be expected on progenies. Butelli et al. (2012) indicated that the red color in the pulp and/or peel of blood orange arose by insertion of a Copia-like retrotransposon adjacent to the gene *Ruby*. In this trait, controlled by a single dominant allele located on terminal region of LG6 (Butelli et al., 2017), the probability to obtain triploid hybrids that inherited this trait is higher using doubled diploid ‘Sanguinelli’ as male parent (66% in heterozygosity plus 16% in dominant homozygosity) in 2x × 4x crosses than in 2x × 2x crosses using diploid “Sanguinelli” also as male parent (50% considering a normal Mendelian segregation manner). Unreduced pollen gametes have been described in several citrus genotypes (Rouiss et al., 2017), and it does not seem to be a general phenomenon. Rouiss et al. (2017) reported the coexistence of two meiotic restitution mechanisms (SDR and FDR) producing unreduced pollen gametes, and the average of PHR for FDR-2n gamete population was 88%, whereas for SDR-2n gamete was 30%. Thus, with the objective of developing new triploid hybrids with this trait, exploitation of 2n-FDR pollen in diploid crosses should be the best strategy (88% of PHR plus 6% in homozygosity), followed by 2x × 4x crosses with doubled diploid “Sanguinelli” as male parent, even though 2n-pollen gametes in citrus is not a frequent event.

Summarizing, in citrus, this is the first deep study conducted on the pollen ontogeny of diploid and doubled diploid “Clemenules” clementine and “Sanguinelli” blood orange. Significant differences in flower size were observed in relation to ploidy level, with the flower size of doubled diploid being larger than that of diploids across two genotypes. The doubled diploids also had a larger pollen cell area, except for “Clemenules,” which presented a dramatic loss of area and pollen shrinkage. During microsporogenesis, no differences were found between genotypes and ploidy levels, except for the larger MiMC area of the doubled diploid genotypes. However, during microgametogenesis, important differences were observed, especially after the release of doubled diploid “Clemenules” clementine microspores, showing that the loss of pollen fertility is due to the failure of pollen to accumulate carbohydrates during microgametogenesis, especially starch, leading to pollen grain abortion. All these changes make the pollen of doubled diploid “Clemenules” clementine unviable, and very difficult to use as a male parent in sexual hybridization with the aim of recovering large progenies of citrus triploid hybrids.

## Conclusion

In this study, we deeply characterized the microsporogenesis and microgametogenesis processes, as well as pollen wall formation, based on light microscope histological preparations and inmunocytochemistry, which have not been described previously in citrus. The ploidy level of pollen grains is not the only factor that reduces their viability, since doubled diploids “Sanguinelli” blood orange and “Clemenules” clementine display the same ploidy but have different pollen performances, suggesting that there is a difference between them during the process of pollen development. We revealed that this difference is not present during microsporogenesis where all the genotypes present normal meiosis and cytokinesis, but during microgametogenesis, as it was observed on double diploid “Clemenules,” which did not present starch grains after vacuolization of the microspores. Moreover, mitosis I was not observed. These issues led to shrinkage of the microspores after vacuolization, resulting in abortion. Therefore, double diploid “Clemenules” is very difficult to be used as a male parent for obtention of large triploid progenies in citrus breeding programs. In the near future, this study will be performed on other double diploid genotypes existing in our collection recovered with antimitotic chemicals or by spontaneous chromosome doubling to further study the effect of polyploidy and the methodology used for their obtention on pollen performance.

## Data Availability Statement

The original contributions presented in the study are included in the article/Supplementary Material, further inquiries can be directed to the corresponding author/s.

## Author Contributions

PA conceived and designed the experiment. JL and AG-L conducted the experiments. JL, AG-L, and PA wrote, reviewed, and edited the manuscript. All authors contributed to the article and approved the submitted version.

## Conflict of Interest

The authors declare that the research was conducted in the absence of any commercial or financial relationships that could be construed as a potential conflict of interest.

## Publisher’s Note

All claims expressed in this article are solely those of the authors and do not necessarily represent those of their affiliated organizations, or those of the publisher, the editors and the reviewers. Any product that may be evaluated in this article, or claim that may be made by its manufacturer, is not guaranteed or endorsed by the publisher.

## Supplementary Material

Supplementary Figure 1 Pollen grains stained with aniline blue and identification of the flower state in which they were observed. **(A)** Diploid “Clemenules,” 3-mm-long flower buds. **(B)** Doubled diploid “Clemenules,” 6-mm-long flower buds. **(C)** Diploid “Sanguinelli,” 8-mm-long flower buds. **(D)** Doubled diploid “Sanguinelli,” 9-mm-long flower buds.

Supplementary Figure 2 Pollen grain germination of diploid and doubled diploid “Clemenules” clementine and “Sanguinelli” blood orange on the “Fortune” mandarin stigma surface. **(A)** Diploid “Clemenules”, **(B)** doubled diploid “Clemenules”, **(C)** diploid “Sanguinelli,” and **(D)** doubled diploid “Sanguinelli.” Pollen grains (pg) and pollen tubes (pt) are marked by arrows. Stigma surface (sf).

Supplementary Figure 3 Inmunolocalization of pectins during microsporogenesis in “Clemenules” clementine. monoclonal antibody used: JIM5 against unesterified pectins. **(A,E)**. Unesterified pectins present in the remaining part of the microspore mother cell (MiMC) wall and the surrounding tapetum cell wall. **(B,F)** Later during development, a JIM5 fluorescence signal was also observed in the early microspore wall (arrows). **(C,G)** After the release of microspores from the tetrads, a JIM5 fluorescence signal was observed in the aperture sites (arrows) and in the early intine. **(D,H)** When the size of vacuolate microspores increased, there was a weak presence of unesterified pectins. The exine was observed in blue due to autofluorescence, and it is indicated with blue arrows. All scale bars = 25 μm. Tapetum (t), MiMC (m), diploid (2x), and doubled diploid (4x).

Supplementary Figure 4 Inmunolocalization of pectins during microsporogenesis in diploid and doubled diploid “Sanguinelli” blood orange. Monoclonal antibody used: JIM5 against unesterified pectins. **(A,C)** Unesterified pectins present in the remaining part of the microspore mother cell (MiMC) wall. **(B,D)** Later during development, unesterified pectins were observed in the aperture sites (asterisks in upper inset) and on the early microspore wall (arrows). All scale bars = 25 μm. Tapetum (t), MiMC (m), diploid (2x), and doubled diploid (4x).
