# Supplementary material for: Pollen Development and Viability in Diploid and Doubled Diploid Citrus Species
Source: Front Plant Sci. 2022 Apr 25;13:862813. doi: 10.3389/fpls.2022.862813 (PMC9090487; doi:10.3389/fpls.2022.862813)
Supplement: Supplementary file 1 [file Table_1.docx]

| **Supplementary material Table 1. Two proportion Z-test for *in vitro* and *in vivo* pollen germination percentages of diploid and doubled diploid `Clemenules´ clementine and `Sanguinelli´ blood orange.** | | | | | | | | |
| --- | --- | --- | --- | --- | --- | --- | --- | --- |
|  | Clem 2x  In vitro | Clem 2x  In vivo | Clem 4x  In vitro | Clem 4x  In vivo | Sang 2x  In vitro | Sang 2x  In vivo | Sang 4x  In vitro | Sang 4x  In vivo |
| Clem 2x  In vitro |  |  |  |  |  |  |  |  |
| Clem 2x  In vivo | 0.0002 |  |  |  |  |  |  |  |
| Clem 4x  In vitro | 0.0000 | 0.0000 |  |  |  |  |  |  |
| Clem 4x  In vivo | 0.0000 | 0.0000 | **0.8874** |  |  |  |  |  |
| Sang 2x  In vitro | 0.0000 | 0.0000 | 0.0004 | 0.0003 |  |  |  |  |
| Sang 2x  In vivo | 0.0000 | 0.0006 | 0.0001 | 0.0000 | 0.0012 |  |  |  |
| Sang 4x  In vitro | 0.0000 | 0.0000 | 0.0002 | 0.0002 | **0.8506** | 0.0021 |  |  |
| Sang 4x  In vivo | 0.0000 | 0.0000 | 0.0000 | 0.0000 | **0.0855** | **0.1142** | **0.1245** |  |
| Clem 2x, Diploid Clemenules; Clem 4x, Doubled diploid Clemenules; Sang 2x, Diploid Sanguinelli; Sang 4x, Doubled diploid Sanguinelli. | | | | | | | | |
| Two proportion Z-test with 5% of significance level (α = 0.05) and considering binomial proportions. Null hypothesis: P1 = P2. The alternative hypothesis: P1 ≠ P2. | | | | | | | | |
| When the p-value is less than significance level of 0.05, we reject the null hypothesis. P-values highlighted in bold corresponds with no statistical differences, the null hypothesis is not rejected. | | | | | | | | |
