# Supplementary figures and images for: Pollen Development and Viability in Diploid and Doubled Diploid Citrus Species
Source: Front Plant Sci. 2022 Apr 25;13:862813. doi: 10.3389/fpls.2022.862813 (PMC9090487; doi:10.3389/fpls.2022.862813)

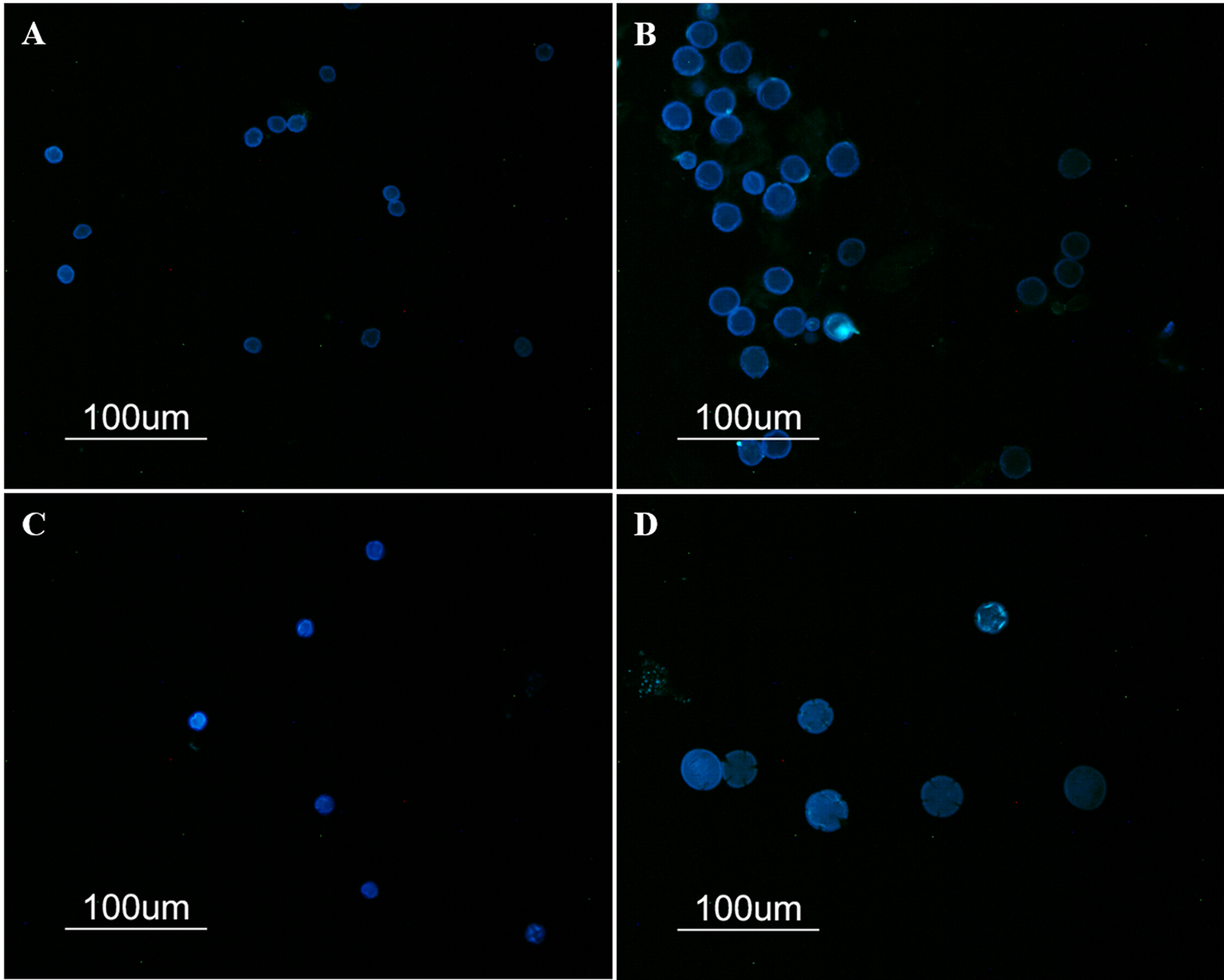

Supplement: Supplementary Figure 1 — Pollen grains stained with aniline blue and identification of the flower state in which they were observed. (A) Diploid “Clemenules,” 3-mm-long flower buds. (B) Doubled diploid “Clemenules,” 6-mm-long flower buds. (C) Diploid “Sanguinelli,” 8-mm-long flower buds. (D) Doubled diploid “Sanguinelli,” 9-mm-long flower buds. [file Image_1.TIF]

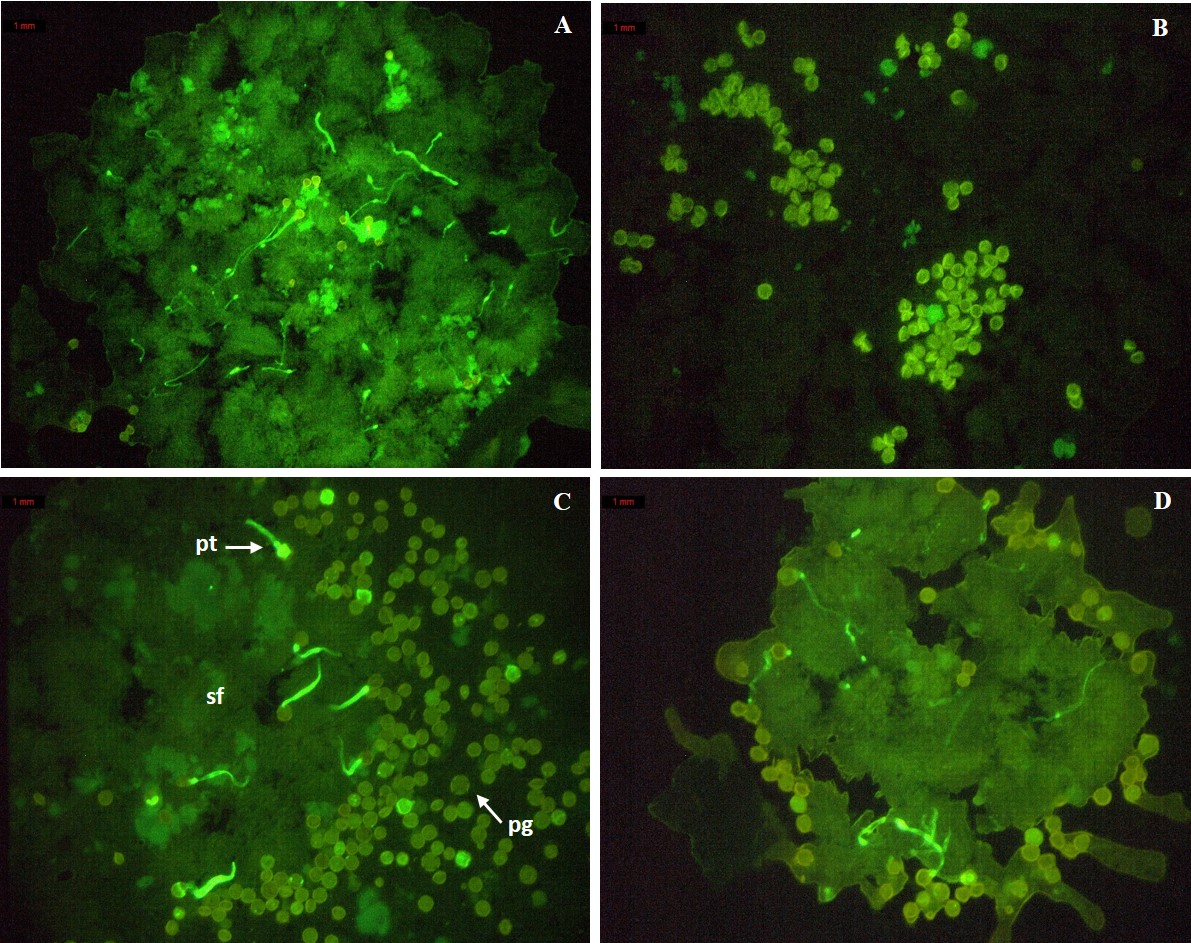

Supplement: Supplementary Figure 2 — Pollen grain germination of diploid and doubled diploid “Clemenules” clementine and “Sanguinelli” blood orange on the “Fortune” mandarin stigma surface. (A) Diploid “Clemenules”, (B) doubled diploid “Clemenules”, (C) diploid “Sanguinelli,” and (D) doubled diploid “Sanguinelli.” Pollen grains (pg) and pollen tubes (pt) are marked by arrows. Stigma surface (sf). [file Image_2.jpg]

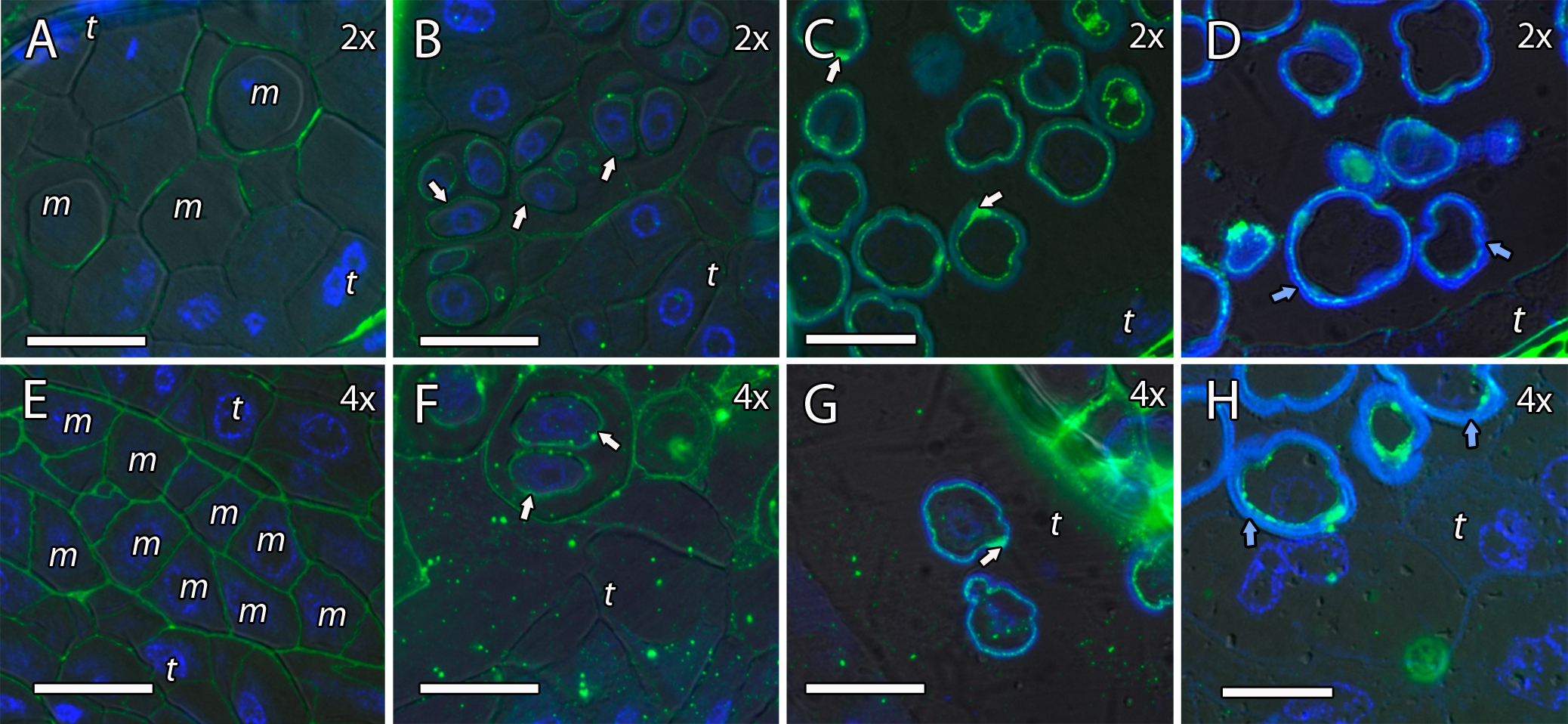

Supplement: Supplementary Figure 3 — Inmunolocalization of pectins during microsporogenesis in “Clemenules” clementine. monoclonal antibody used: JIM5 against unesterified pectins. (A,E). Unesterified pectins present in the remaining part of the microspore mother cell (MiMC) wall and the surrounding tapetum cell wall. (B,F) Later during development, a JIM5 fluorescence signal was also observed in the early microspore wall (arrows). (C,G) After the release of microspores from the tetrads, a JIM5 fluorescence signal was observed in the aperture sites (arrows) and in the early intine. (D,H) When the size of vacuolate microspores increased, there was a weak presence of unesterified pectins. The exine was observed in blue due to autofluorescence, and it is indicated with blue arrows. All scale bars = 25 μm. Tapetum (t), MiMC (m), diploid (2x), and doubled diploid (4x). [file Image_3.JPEG]

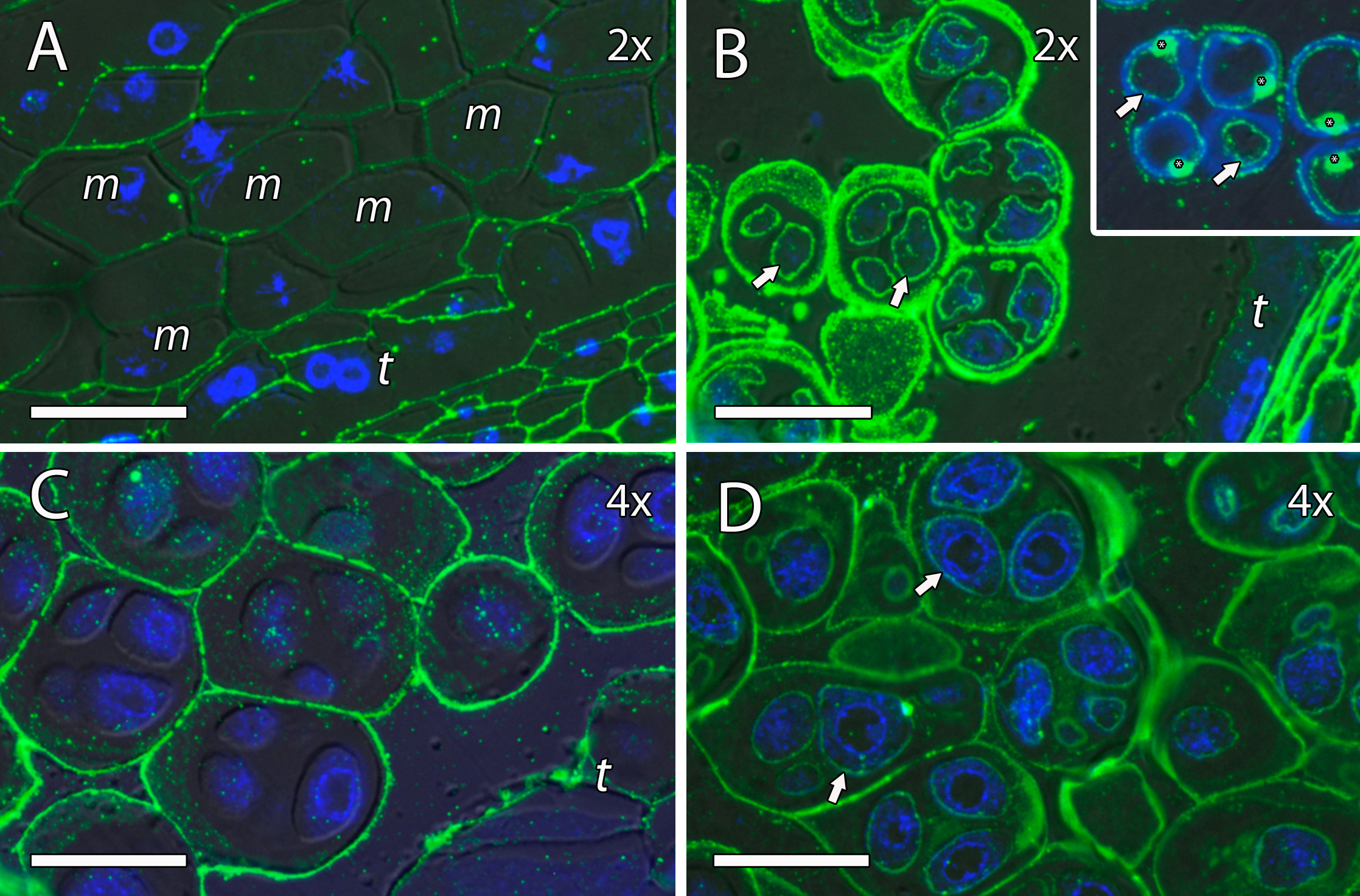

Supplement: Supplementary Figure 4 — Inmunolocalization of pectins during microsporogenesis in diploid and doubled diploid “Sanguinelli” blood orange. Monoclonal antibody used: JIM5 against unesterified pectins. (A,C) Unesterified pectins present in the remaining part of the microspore mother cell (MiMC) wall. (B,D) Later during development, unesterified pectins were observed in the aperture sites (asterisks in upper inset) and on the early microspore wall (arrows). All scale bars = 25 μm. Tapetum (t), MiMC (m), diploid (2x), and doubled diploid (4x). [file Image_4.JPEG]
